# Epidemiology, resistance profiles, and molecular characteristics of pediatric salmonella isolates: prevalence of a clonal *bla_NDM-5_*-bearing *S.* typhimurium ST34 cluster in Nanning

**DOI:** 10.3389/fcimb.2026.1931024

**Published:** 2026-09-03

**Authors:** Zhenjiao Cen, Wenwen Qiu, Jialing Ruan, Shangjuan Zeng, Jiahui Liang, Huan Zhang, Chenglan Yan, Linlin Li

**Affiliations:** 1 Department of Clinical Laboratory, Children’s Hospital, Maternal and Child Health Hospital of Guangxi Zhuang Autonomous Region, Nanning, China; 2 Department of Maternal and Fetal Medicine, Children’s Hospital, Maternal and Child Health Hospital of Guangxi Zhuang Autonomous Region, Nanning, China

**Keywords:** blaNDM-5, carbapenem-resistant, pediatric, *S. Typhimurium*, salmonella

## Abstract

**Background:**

*Salmonella* poses a significant threat to immunocompromised patients and is associated with high morbidity and mortality rates. Pediatric populations, owing to their immature immune systems and lack of specific protective antibodies, are particularly susceptible to *Salmonella* infection. This study aimed to comprehensively investigate the epidemiology, resistance profiles, and molecular characteristics of pediatric *Salmonella* isolates.

**Methods:**

A retrospective study was conducted at Guangxi Children’s Hospital, in Nanning, China, spanning from January to December 2025. Isolates were identified by MALDI-TOF MS and serotyped via slide agglutination. Antibiotic susceptibility testing was performed using either the DL-96 II or VITEK 2 Compact system. Whole-genome sequencing (WGS) was conducted on the 11 identified carbapenem-resistant *Salmonella* isolates. Genomic analyses included multilocus sequence typing (MLST), serotyping, virulence and antimicrobial resistance gene profiling, and SNP-based phylogenetic analysis.

**Results:**

Among 1,372 unique *Salmonella* isolates, *S.* Typhimurium was the predominant serotype (55.1%, 757/1,372), peaking in infants aged less than 1 year (n = 213) and during late spring and summer (May-June). High resistance rates were observed for ampicillin (70.1%), chloramphenicol (49.1%), sulfamethoxazole (41.0%), and minocycline (39.4%), with *S.* Typhimurium exhibiting significantly higher resistance to multiple antibiotics (including ceftriaxone and carbapenems) than non-Typhimurium strains (*p* < 0.05). Genomic analysis of the 11 carbapenem-resistant *Salmonella* isolates revealed that 10 (90.9%) carried *bla_NDM-5_*, while one carried *bla_NDM-13_*, typically co-existing with *bla_TEM-1B_*, *bla_OXA-10_*, and IncHI2/IncHI2A/IncQ1 replicons. All carbapenem-resistant *Salmonella* isolates shared 111 virulence genes (across 17 categories), showing a 100% carriage rate for 83 core virulence genes. Furthermore, *S.* Typhimurium ST34 carrying *bla_NDM-5_* was identified as the predominant genetically related lineage.

**Conclusion:**

Pediatric *Salmonella* infections, predominantly caused by *S.* Typhimurium, exhibit strong seasonality and primarily affect children under 2 years of age. Importantly, the detection of this genetically related, *bla_NDM-5_*-positive *S.* Typhimurium ST34 lineage among pediatric patients underscores the risk of potential clonal dissemination within the clinical setting. These findings underscore the urgent need for continuous genomic surveillance of carbapenem-resistant *Salmonella* in pediatric settings.

## Introduction

As a major Gram-negative, zoonotic pathogen, *Salmonella* poses a significant threat to global food safety and public health (Espinoza-Erazo et al., 2025). The genus comprises two primary species: *S.* enterica—which is further classified into six subspecies and nearly 3,000 serovars—and *S.* bongori (Worley, 2023). In humans, infection typically manifests as gastroenteritis, potentially leading to severe diarrhea and mortality, particularly in pediatric populations (Riaz et al., 2025). Globally, non-typhoidal *Salmonella* (NTS) is conservatively estimated to cause approximately 93.8 million gastroenteritis cases and 155,000 deaths annually (Majowicz et al., 2010). The World Health Organization has designated fluoroquinolone-resistant *Salmonella* as high-priority pathogens on the list of antibiotic-resistant bacteria (Sati et al., 2025). This pathogen harbors a diverse array of virulence genes within characterized *Salmonella* pathogenicity islands (SPIs), which mediate host cell attachment, invasion, and survival (Woh et al., 2025). Crucially, the co-selection and synergism between these virulence determinants and antimicrobial resistance genes further compound the clinical challenge of treating infections.

Although NTS infection is typically self-limiting, it can progress to severe systemic disease in immunocompromised individuals, such as children and the elderly, thereby necessitating antibiotic therapy (Park et al., 2021). In recent years, the reliance on fluoroquinolones and broad-spectrum cephalosporins as primary treatment options for NTS has led to a rise in resistance rates among NTS strains (Chowdhury et al., 2022; Kuehn et al., 2025). *Salmonella* enterica serovar Typhimurium (*S*. Typhimurium) is one of the most frequent serotypes and can exhibit resistance to a wide range of antibiotics, including well-regarded ones (Sun et al., 2020). Antibiotic resistance significantly plays an important role in the rising incidence of various bacterial infections. The high prevalence of resistance and multidrug resistance (MDR) in *S.* Typhimurium, reported across various nations, underscores a major driver behind the widespread dissemination of this serovar (Chiou et al., 2023; Van Puyvelde et al., 2023; Ali et al., 2024). The emergence and dissemination of widespread antibiotic resistance in *Salmonella* are primarily driven by the use and misuse of antibiotics in both humans and animals, including livestock and aquaculture (Sabeq et al., 2022; Yin et al., 2022; Matle et al., 2025). Carbapenems are broad-spectrum β-lactam antibiotics indicated for severe bacterial infections, rendering them the last resort for managing aggressive, multidrug-resistant NTS infections (Wu et al., 2023; Global burden of bacterial antimicrobial resistance 1990-2021: A systematic analysis with forecasts to 2050, 2024). Despite the rare occurrence of carbapenem-resistant *S.* Typhimurium, its emergence, largely driven by *bla_NDM_* gene dissemination, poses a critical threat to antimicrobial therapy (Song et al., 2023; Ke et al., 2024). Currently, the phylogenetic structure and epidemiological dynamics of these resistant strains remain poorly understood.

The evolutionary lineage and resistome of *Salmonella* undergo continuous spatiotemporal variations, resulting in distinct regional characteristics (Feng et al., 2025). Accordingly, heightened concerns regarding drug-resistant bacterial enteritis in pediatric have positioned *Salmonella* as an important public health concern in endemic regions like Southwest China. Therefore, the primary objective of this study was to investigate the prevalence of *Salmonella* in stool cultures of pediatric patients presenting at a tertiary care children’s hospital. Additionally, this study aimed to identify *Salmonella* serotypes, analyze their antimicrobial resistance patterns, and examine demographic (age and gender) and seasonal variations over a one-year period. Furthermore, we sought to characterize the molecular epidemiology of carbapenem-resistant *Salmonella* isolates by profiling their resistance genes, sequence types, virulence determinants, and plasmid replicons.

## Methods

### Study setting and patients

**Figure 1:**
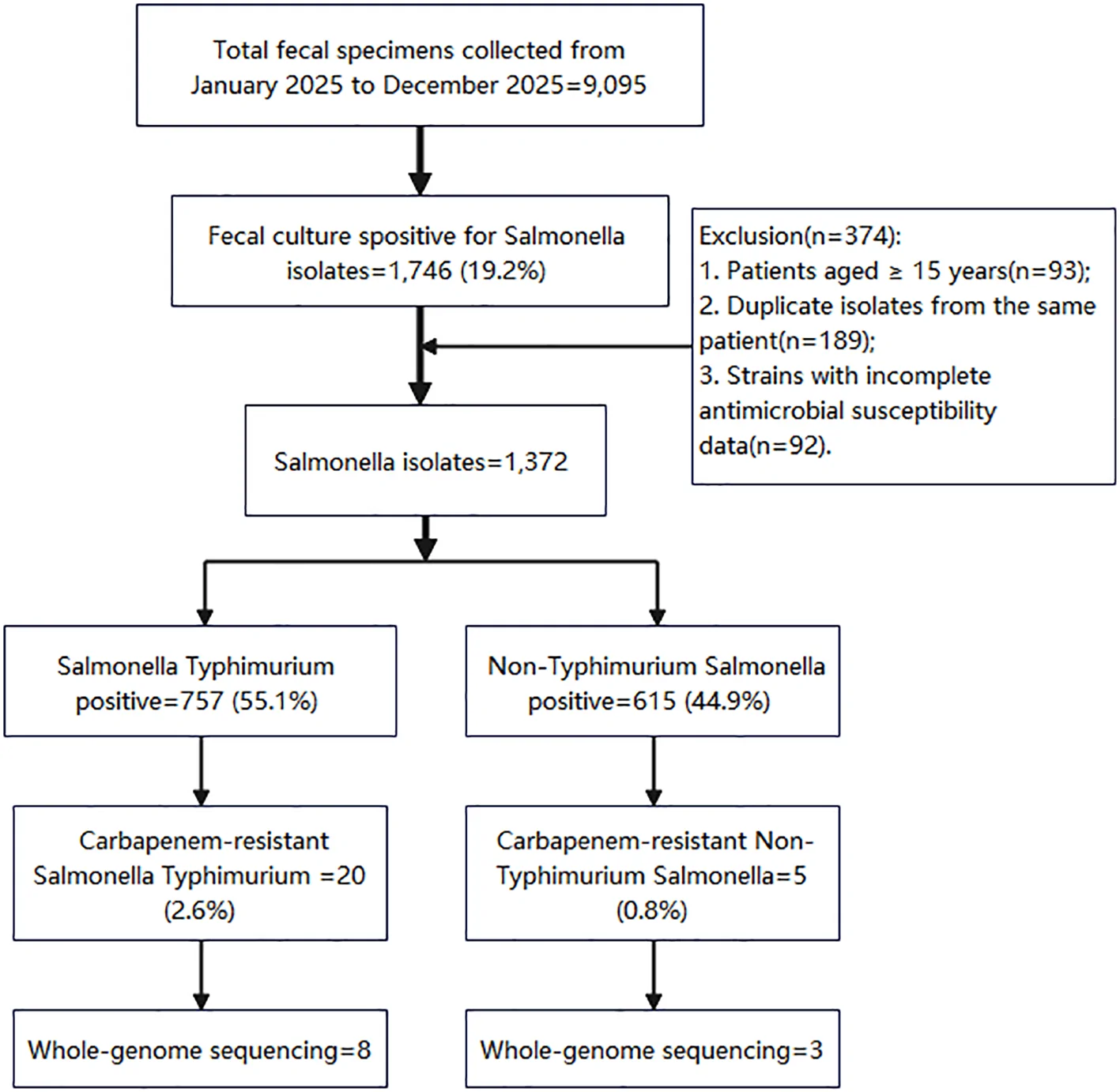
Flowchart for enrolled patients.

A retrospective study was conducted at the Guangxi Zhuang Autonomous Region Maternal and Child Health Hospital, which is the largest maternity and pediatric care center in the region. From January through December of 2025, a total of 9,095 fecal specimens were collected, and among these, 1,746 (19.2%) tested positive for *Salmonella*, as shown in **Figure 1**. Participants were categorized into four age groups based on months of age: <1 year (0–11 months), 1 year (12–23 months), 2–3 years (24–47 months), and 4–14 years (48 months to 14 years). Specifically, ‘1 year’ refers to children who have reached their first birthday but have not yet reached their second. In this study, only the first *Salmonella* isolate from fecal cultures was analyzed for each patient. The inclusion criteria included the following: (a) patients aged less than 15 years; (b) complete antimicrobial susceptibility data and complete demographic data. Exclusion Criteria: (a) patients aged greater than or equal to 15 years; (b) duplicate isolates from the same patient; and (c) strains with incomplete antimicrobial susceptibility data. A total of 1,372 unique *Salmonella* isolates were included in the final analysis. This study was divided into two main groups based on serotypes: *S.* Typhimurium and non-Typhimurium *Salmonella* strains. Due to limited research funding, WGS was employed to perform an exploratory genomic analysis of 11 of the 25 carbapenem-resistant *Salmonella* isolates. To minimize potential selection bias, a pre-specified, structured sampling strategy was employed to select the 11 isolates for WGS. Specifically, these isolates were selected to represent: (1) temporal distribution across the entire study period of 2025 to avoid clustering in a single outbreak event; (2) patient age diversity (spanning various age groups in months); and (3) varied clinical settings, encompassing both outpatient and inpatient departments. The study protocol was approved by the research administration of the Medical Ethics Committee at the Maternal and Child Health Hospital of Guangxi Zhuang Autonomous Region (with Ethics ID: 2026-3-29).

### Bacterial isolates and antimicrobial susceptibility

Extract a sufficient amount of fecal sample and inoculate it onto blood agar and MacConkey agar plates using an inoculating loop. Meanwhile, use a cotton swab to take a sufficient amount of feces and inoculate it into *Salmonella* enrichment broth for enrichment. After 24 hours of enrichment, subculture the broth onto an SS (*Salmonella*-Shigella) agar plate. On SS agar, typical *Salmonella* colonies appear colorless and translucent with a black center. Following incubation at 37 °C for 16–18 hours, a single colony was picked and subcultured onto a blood agar plate to obtain pure isolates for subsequent identification and antimicrobial susceptibility testing. Isolates were identified by matrix-assisted laser desorption ionization time-of-flight mass spectrometry (MALDI-TOF MS; Autof MS6000, China). *Salmonella* serotyping was performed by slide agglutination using specific antisera (Tianrun, Ningbo, China) according to the manufacturer’s instructions. The antibiotic susceptibility tests were performed on the isolates tested using either the Kirby-Bauer methodology, the Zhuhai DL-96II automated bacterial identification drug sensitivity apparatus, or the VITEK 2 Compact system. Each of these protocols was conducted in accordance with the manufacturer’s instructions. Results were assessed in accordance with the Clinical and Laboratory Standards Institute (CLSI) 2025 guidelines. Multidrug resistance (MDR) was defined as non-susceptibility (including resistance and intermediate susceptibility) to at least one agent in three or more antimicrobial categories, in accordance with established international expert guidelines (Magiorakos et al., 2012). The quality control strain was *Escherichia coli* ATCC 25922 (at the Chinese National Center for Clinical Laboratories).

### Whole genome sequencing and analysis

After assessing antibiotic resistance profiles, the study performed an exploratory genomic analysis via WGS on eleven representative carbapenem-resistant *Salmonella* isolates. Bacterial genomic DNA was isolated using the MagPure Bacterial DNA Kit (D6361-02, Magen, China), and its concentration was subsequently quantified with a Qubit 4.0 fluorometer (Thermo, Q33226). The integrity of the extracted DNA was evaluated via 1% agarose gel electrophoresis. Subsequently, the genomic DNA was randomly fragmented to an average size of 200–400 bp. Library preparation was then performed on the selected fragments, involving end-repair, 3’ adenylation, adapter ligation, and PCR amplification. Following magnetic bead-based purification, library concentration and size distribution were evaluated using a Qubit 4.0 fluorometer and 2% agarose gel electrophoresis, respectively. The resulting high-quality libraries were sequenced on an Illumina NovaSeq 6000 instrument (Sangon Biotech, Shanghai, China) with a 150-bp paired-end configuration, yielding an average sequencing coverage depth of ~98×.

For raw data quality control, raw reads were filtered via Trimmomatic (v0.36) by removing adaptors and low quality reads (reads containing >40% bases with quality <Q15 or reads shorter than 35 bp), then clean reads were obtained.Genome assembly was done using SPAdes(v3.15)and the Gapfiller(v1.11) was used for filling gaps. The quality of the final genome assembly was evaluated using multiple metrics, including the total number of scaffolds, total assembly length, scaffold N50 and N90 sizes, and GC content. The completeness and contamination level of the assembled genome were assessed using CheckM.Coding sequences (CDSs), tRNAs, and rRNAs were predicted using Prokka (v1.10).

Functional annotations were subsequently assigned by aligning the predicted protein sequences against the National Center for Biotechnology Information (NCBI) Non-redundant (NR) database, applying cutoff thresholds of ≥ 90% sequence identity (pident) and ≥80% query coverage (qcov). Additionally, tandemly repeated DNA motifs were identified using Tandem Repeat Finder (TRF, v4.09). Plasmid replicon profiling was conducted using the command-line version of PlasmidFinder and ResFinder (v2.1) with minimum identity of 95% and coverage of 60%. The conjugative modules of the resistance-carrying contigs were analyzed using oriTfinder (v2.1) with default parameters to predict their transferability. Contigs containing the origin of transfer (*oriT*), relaxase, T4CP, and T4SS genes were predicted as fully conjugative. Multilocus sequence typing (MLST) was performed using the MLST tool (v2.19) (https://github.com/tseemann/mlst) against the EnteroBase Salmonella database.

For phylogenomic analysis, single-nucleotide polymorphisms (SNPs) were identified by mapping clean reads against the reference genome of *Salmonella* enterica serovar Typhimurium strain LT2 (GenBank accession no. AE006468) using Snippy (v4.6.0). High-quality SNPs were called using the following parameters: a minimum mapping quality of 60, a minimum alignment depth of 10×, and a consensus allele frequency of ≥90%. Core genome SNP alignment was constructed, and recombinant regions were filtered. Based on the identified core SNPs, a neighbor-joining phylogenetic tree was reconstructed using FastTree and was subsequently visualized using iTOL (v6).

### Statistical analysis

Statistical analyses were performed using SPSS version 25.0. Categorical variables were expressed as frequencies (%) and analyzed using the χ² tests or Fisher’s exact test as appropriate. Visualization of serotypes, antibiotic resistance, virulence factors, and plasmids was performed using the online tool ChiPlot (https://www.chiplot.online/). 

## Results

### Demographic distribution of *Salmonella* isolates

**Figure 2:**
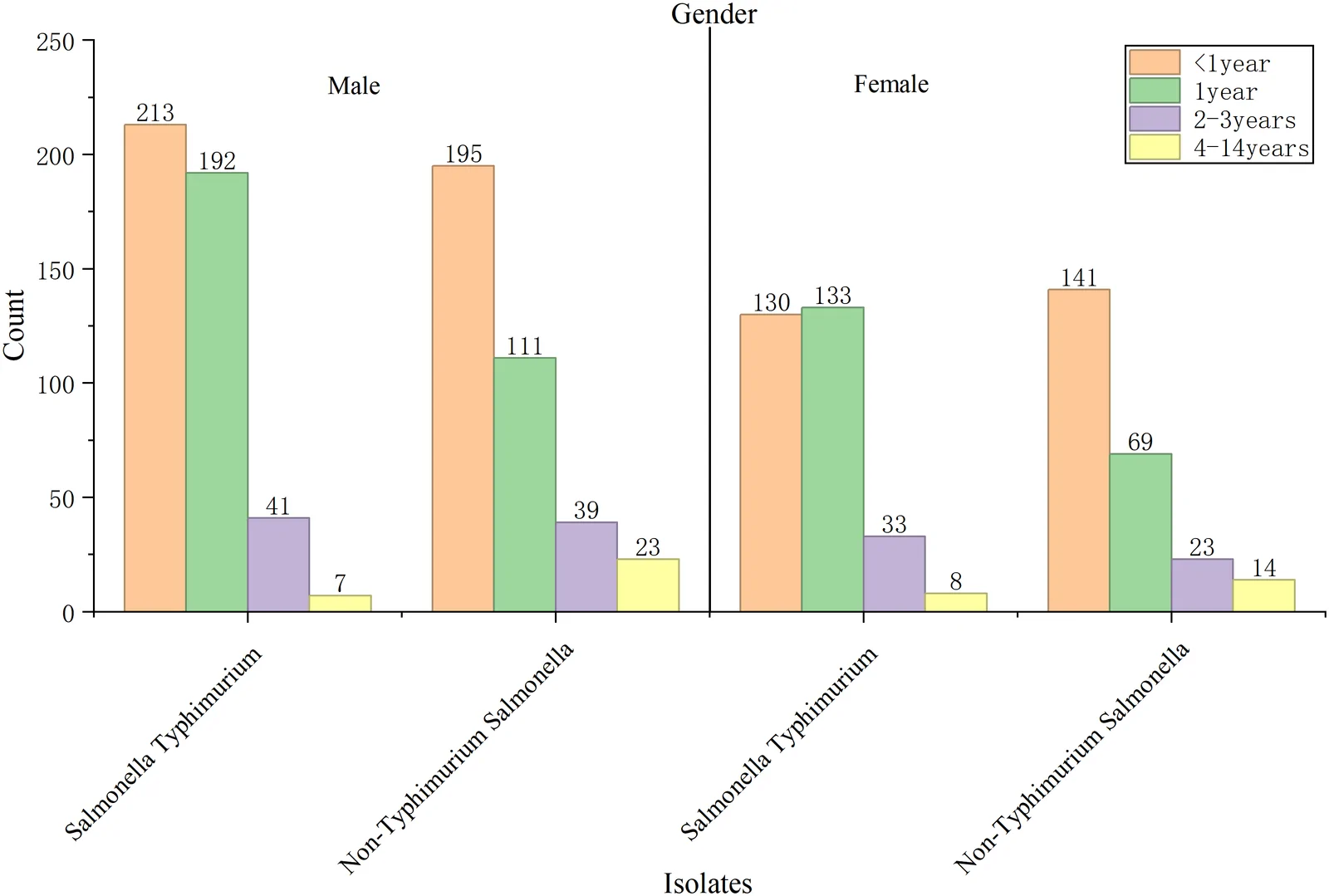
Demographic distribution of *S.* Typhimurium and non-Typhimurium *Salmonella* isolates. The isolates are stratified by gender (male vs. female) and age groups (<1 year, 1 year, 2–3 years, and 4–14 years). The numbers above the bars indicate the count of isolates in each category.

During the study period, 1,372 unique *Salmonella* isolates were enrolled. Among these, *S.* Typhimurium was the predominant serotype, accounting for 55.1% (757/1372) of the isolates, while non-Typhimurium *Salmonella* accounted for 44.9% (615/1372). **Figure 2** shows the distribution of *Salmonella* isolates stratified by gender, age groups, and serovar categories. In male patients, a higher burden of infection was observed compared to female patients. Across both genders and isolate types, the prevalence was heavily concentrated in infants under 1 year of age and those aged 1 year. There was a sharp decline in older age groups, specifically in the 2–3 years and 4–14 years age groups. Specifically, among male patients, cases of *S.* Typhimurium peaked in infants less than 1 year old (n = 213), followed closely by those aged 1 year (n = 192). A similar age-related pattern was observed for male non-Typhimurium *Salmonella* isolates, which peaked at less than 1 year (n = 195). This was followed by a decrease to a total of 111 cases in the 1-year-old group. Among female patients, *S.* Typhimurium cases were similar between the less than 1 year old (n = 130) and 1-year-old (n = 133) groups. For non-Typhimurium *Salmonella* in females, the highest caseload was also recorded in the less than 1 year old cohort (n = 141), which was more than double the caseload of the 1-year-old cohort (n = 69).

### Seasonal distribution of *Salmonella* isolates

**Figure 3:**
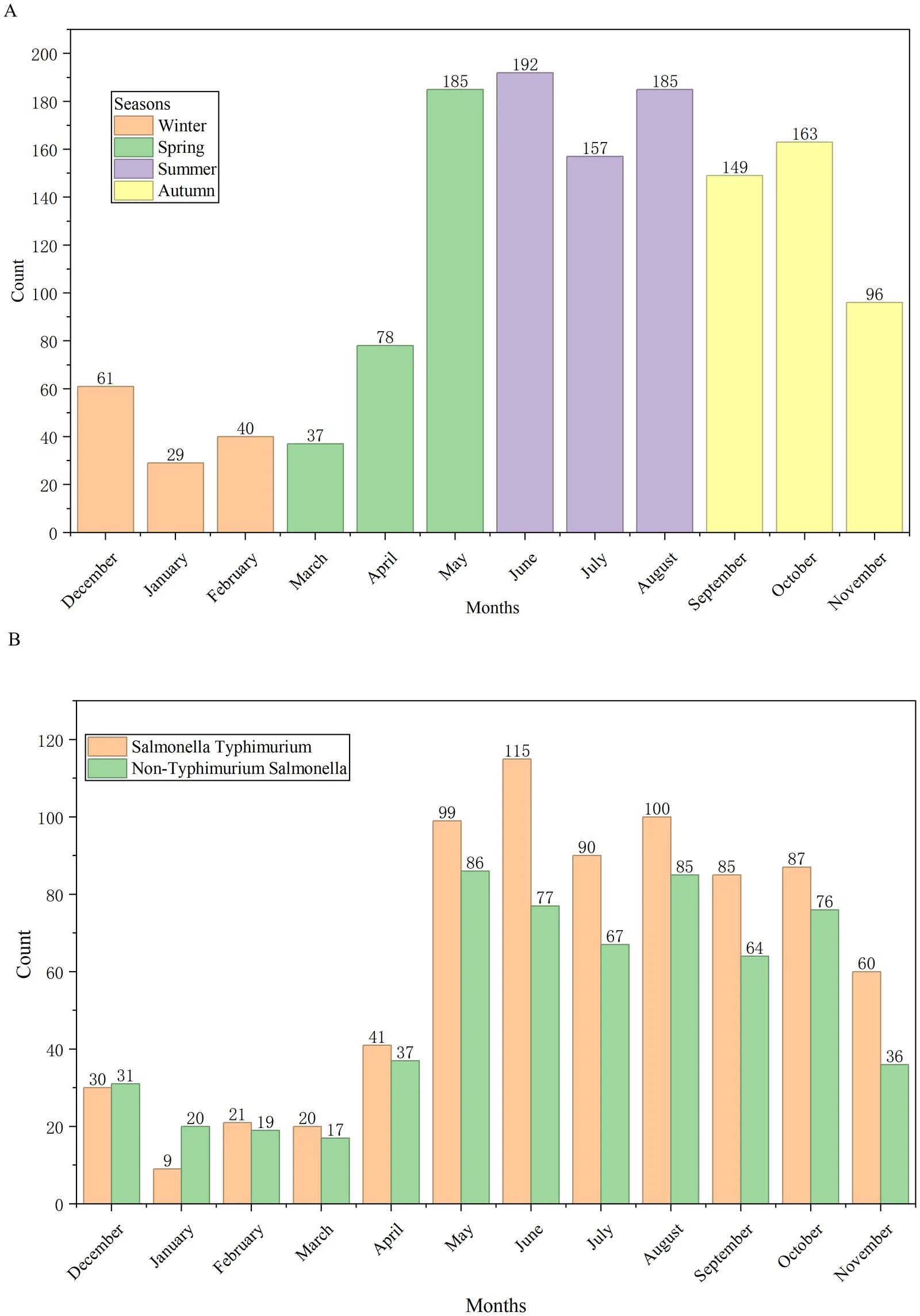
Monthly and seasonal distribution of the collected isolates. **(A)** Total number of *Salmonella* isolates collected by month and season. **(B)** Monthly distribution comparison between *S.* Typhimurium and non-Typhimurium *Salmonella* isolates.

Temporal analysis revealed a distinct seasonal variation in *Salmonella* prevalence. The incidence was relatively low during the winter months, with the lowest counts recorded in January (n=29). The isolation rate peaked during the summer and late spring, with the highest number of cases recorded in June (n = 192) and May (n = 185) (**Figure 3A**). This surge in incidence continued through the summer and early autumn, before declining in November (n=96). Notably, both *S.* Typhimurium and non-Typhimurium *Salmonella* followed similar seasonal trends, the number of *S.* Typhimurium cases consistently exceeded that of non-Typhimurium *Salmonella* throughout most of the study period. The discrepancy between the two groups peaked during the high-transmission season, with the widest gap recorded in June (115 *S.* Typhimurium vs. 77 non-Typhimurium *Salmonella*) (**Figure 3B**).

### Antimicrobial susceptibility of *Salmonella* isolates

1,372 *Salmonella* isolates were analyzed for the antimicrobial susceptibility to 10 commonly used antimicrobial agents (**Table 1**). The results indicated that high resistance rates were observed for ampicillin (70.1%), chloramphenicol (49.1%), sulfamethoxazole (41.0%), and minocycline (39.4%). Conversely, resistance rates to carbapenems (imipenem and meropenem, both 1.8%), cefoperazone-sulbactam (3.5%), and fluoroquinolones (levofloxacin, 4.3%; ciprofloxacin, 6.9%) remained relatively low. Additionally, our results revealed that resistance rates to minocycline, ampicillin, sulfamethoxazole, cefoperazone-sulbactam, ceftriaxone, imipenem, meropenem, and chloramphenicol were significantly higher in *S.* Typhimurium than in non-Typhimurium *Salmonella* (*p* < 0.05). Interestingly, carbapenem resistance was identified in 2.6% (20/757) of *S.* Typhimurium isolates and in 0.8% (5/615) of the non-Typhimurium *Salmonella* isolate*s.*

**Table 1:** Antimicrobial resistance profiles of *Salmonella* isolates and comparison between *S.* Typhimurium and non-Typhimurium serotypes.

Antibiotic agent	Salmonella isolates (n=1372) n%	Salmonella typhimurium (n=757) n%	Non-typhimurium salmonella (n=615) n%	*P*-value
Minocycline	541(39.4)	458(60.5)	83(13.4)	**<0.001**
Ampicillin	963(70.1)	681(89.9)	282(45.8)	**<0.001**
Sulfamethoxazole	563(41.0)	389(51.3)	174(28.2)	**<0.001**
Cefoperazone-Sulbactam	49(3.5)	39(5.1)	10(1.6)	**<0.001**
Levofloxacin	60(4.3)	22(2.9)	38(6.1)	**0.003**
Ceftriaxone	258(18.8)	169(22.3)	89(14.4)	**<0.001**
Imipenem	25(1.8)	20(2.6)	5(0.8)	**0.012**
Ciprofloxacin	95(6.9)	44(5.8)	51(8.2)	0.072
Meropenem	25(1.8)	20(2.6)	5(0.8)	**0.012**
Chloramphenicol	674(49.1)	469(61.9)	205(33.3)	**<0.001**

Bold values in the *P*-value column indicated statistical significance.

### Clinical characteristics and antimicrobial susceptibility patterns of carbapenem-resistant *Salmonella* infections

This study analyzed a cohort of 25 pediatric patients infected with carbapenem-resistant *Salmonella* (see **Supplementary Table 1**). The patients ranged in age from 5 months to 3 years, with 17 males and 8 females. In terms of clinical settings, 12 patients (48.0%) were managed at the pediatric outpatient clinic, while 13 (52.0%) were admitted to the Department of Gastroenterology. All 25 bacterial isolates were s recovered from stool specimens. Clinically, pediatric gastroenteritis was diagnosed in all 25 cases. Fecal laboratory investigations revealed positive results for white blood cells in 7 patients (28.0%) and occult blood in 10 patients (40.0%). Ultimately, all patients (100.0%) achieved clinical improvement following therapeutic intervention.

**Figure 4:**
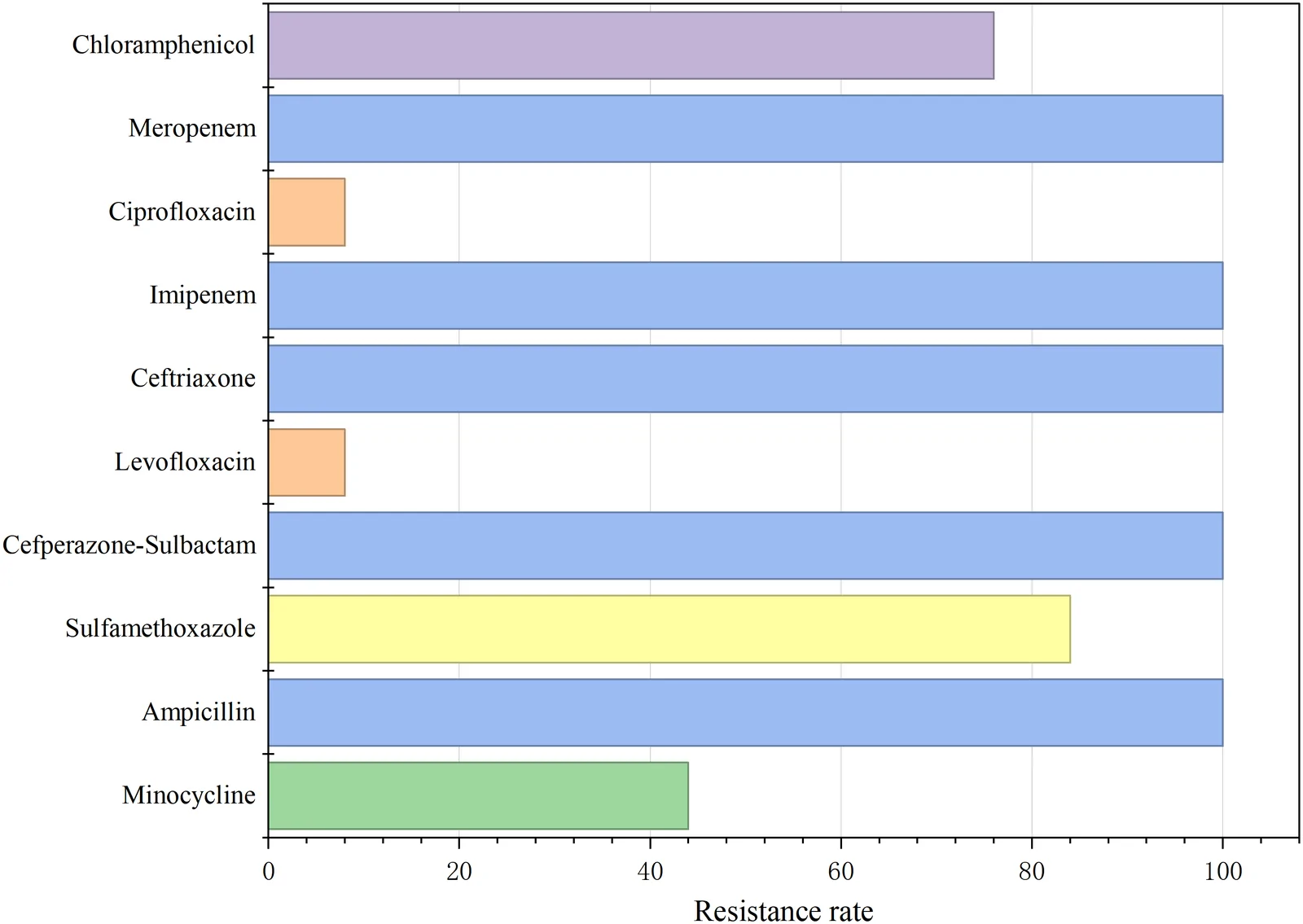
Antimicrobial resistance profiles of 25 carbapenem-resistant *Salmonella* isolates.

Moreover, the antimicrobial susceptibility of the 25 carbapenem-resistant *Salmonella* isolates is shown in **Figure 4**. The results revealed that all 25 isolates were classified as resistant to imipenem, meropenem, ampicillin, ceftriaxone, and cefoperazone-sulbactam. Conversely, these carbapenem-resistant isolates remained highly susceptible to ciprofloxacin and levofloxacin.

### Frequency of AMR genes and plasmid replicons

**Figure 5:**
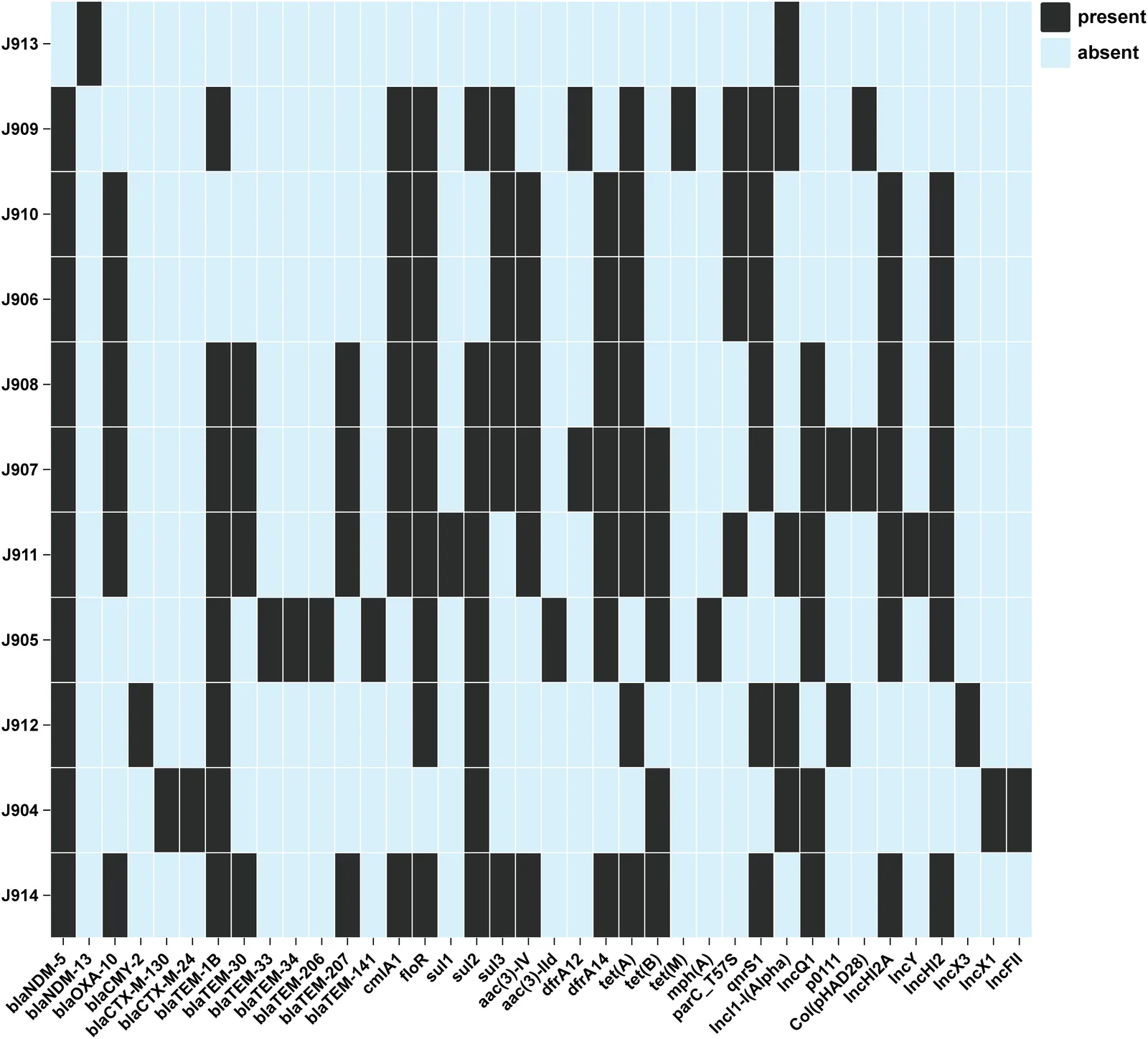
Profiles of antimicrobial resistance genes and plasmid replicons in 11 carbapenem-resistant Salmonella isolates.

Through WGS, eleven carbapenem-resistant *Salmonella* isolates whole-genome sequences were obtained, all with GC contents fixed at 53%. The prevalence and distribution of AMR genes and plasmid replicons among the 11 isolates (J904–J914) were analyzed and visualized in **Figure 5**. Genomic analysis indicated a wide variety of resistance determinants. Regarding beta-lactamase genes, the carbapenemase gene *bla_NDM-5_* was highly prevalent, being detected in 10 out of the 11 isolates (90.9%), whereas *bla_NDM-13_* was uniquely harbored by isolate J913. Other beta-lactamase genes, such as *bla_TEM-1B_* and *bla_OXA-10_*, were also widely distributed. In contrast, *bla_CTX-M-130_*, *bla_CTX-M-24_*, and *bla_CMY-2_* were sparsely identified in specific isolates (e.g., J912 and J904). In addition, non-beta-lactam resistance genes, including *floR* (phenicol resistance), *sul2* (sulfonamide resistance), *aac(3)-IV* (aminoglycoside resistance), and tetracycline resistance determinants (*tet(A)* or *tet(B)*), were highly abundant across the majority of the isolates. Additionally, replicon typing revealed diverse plasmid profiles among the isolates. The IncHI2 and IncHI2A replicons were co-carried by a cluster of isolates (J910, J906, J908, J907, and J911). IncQ1 was another commonly detected replicon. Interestingly, certain replicon types exhibited isolate-specific patterns; for instance, IncX3 was exclusively identified in isolate J912, while IncX1 and IncFII were restricted to isolate J904. Furthermore, only strain J913 (harboring *bla_NDM-13_*) carried the resistance gene on an identifiable plasmid contig (ctg00014), matching the IncI1-I(Alpha) replicon type with 100% identity. Further analysis by oriTfinder predicted this contig to be fully conjugative, as it possessed all essential transfer components: the origin of transfer (oriT), relaxase, Type IV coupling protein (T4CP), and a Type IV secretion system (T4SS). Conversely, for the 10 *bla_NDM-5_*-carrying isolates, no plasmid replicons or conjugative transfer elements were detected on the resistance-bearing contigs (see **Supplementary Table S2** and **Supplementary Figure 1**).

### Frequency of virulence genes

This study performed a systematic analysis of the virulence gene profiles of eleven carbapenem-resistant *Salmonella* strains, which identified a total of 111 virulence genes, including 17 categories (**Table 2**). Among the 83 genes that encompass key pathogenic mechanisms like adhesion and invasion, secretion systems, and immune evasion, a 100% carry rate is observed across all strains. For example, SPI-1 (*avrA, invA, prgK, sopE2*, etc.); SPI-2 (*sopE2, sseF, spiC/ssaB*, etc.); SPI-2 encoded T3SS (*pipB, sscA, sscB*); bovine colonization factors (*bcfA-F*); Agf (*csgC, csgA*, etc.); Type 1 fimbriae (*fimD, fimH, fimC, fimI, fimF*); SPI-1 encoded T3SS (*orgA, orgC*); MgtBC (*mgtB, mgtC*); SinH (*sinH*); RatB (*ratB*); and curli fibers/thin aggregative (*csgD*). In contrast to the other virulence genes, except for *sspH2* (5.0%), *sifB* (63.6%), *pipB2* (63.6%), *steA* (63.6%), *shdA* (63.6%), and *cdtB* (9.1%), the remaining genes have a prevalence rate greater than 70%.

**Table 2:** Virulence gene profiles of the 11 carbapenem-resistant *Salmonella* isolates.

Virulence gene classification	Virulence gene	Carrier rate%
TTSS (SPI-1 encode)	avrA,invA,invB,invC,invE,invF,invG,invH,invI,invJ,orgB, prgH,prgI,prgJ,prgK,sicA,sicP,sipA,sspA, sipB,sspB,sipC,sspC,sipD,sopA,sopB,sigD,sopD, sopE2,spaO,spaP,spaQ,spaR,spaS,sptP	100.0
	slrP	81.8
TTSS (SPI-2 encode)	ssaV,ssaC,ssaN,ssaD,ssaQ,ssaU,ssaL,ssaT,sseF,ssaJ, sseG,ssaK,ssaR,sseE,spiC/ssaB,ssaM,ssaP, ssaO,sseA,ssaS,ssaI,ssaE,ssaH,ssaG	100.0
	sseJ,sseD,sseB	90.9
	sseC,sifA	72.7
	sspH2	5.0
SPI-2 encoded T3SS	pipB,sscA,sscB	100.0
	sseK1	90.9
	sseK2,sopD2,gogB	81.8
	steC,sseL,sseI/srfH	72.7
	sifB,pipB2	63.6
bovine colonization factor (Bcf)	bcfC,bcfD,bcfG,bcfB,bcfE,bcfF,bcfA	100.0
Agf	csgG,csgF,csgE,csgC,csgA,csgB	100.0
Type 1 fimbriae	fimD,fimH,fimC,fimI,fimF	100.0
Lpf	lpfC,lpfB,lpfE,lpfA	81.8
	lpfD	72.7
SPI-1 encoded T3SS	orgA,orgC	100.0
	steA	63.6
MgtBC	mgtB,mgtC	100.0
SinH	sinH	100.0
RatB	ratB	100.0
SodCI	sodCI	72.7
MisL	misL	90.9
Mig-14	mig-14	90.9
curli fibers/thin aggregative fimbriae (AGF)	csgD	100.0
ShdA	shdA	63.6
CdtB	cdtB	9.1

### MLST, serotyping, and SNP phylogenetic tree of carbapenem-resistant *Salmonella* isolates

**Figure 6:**
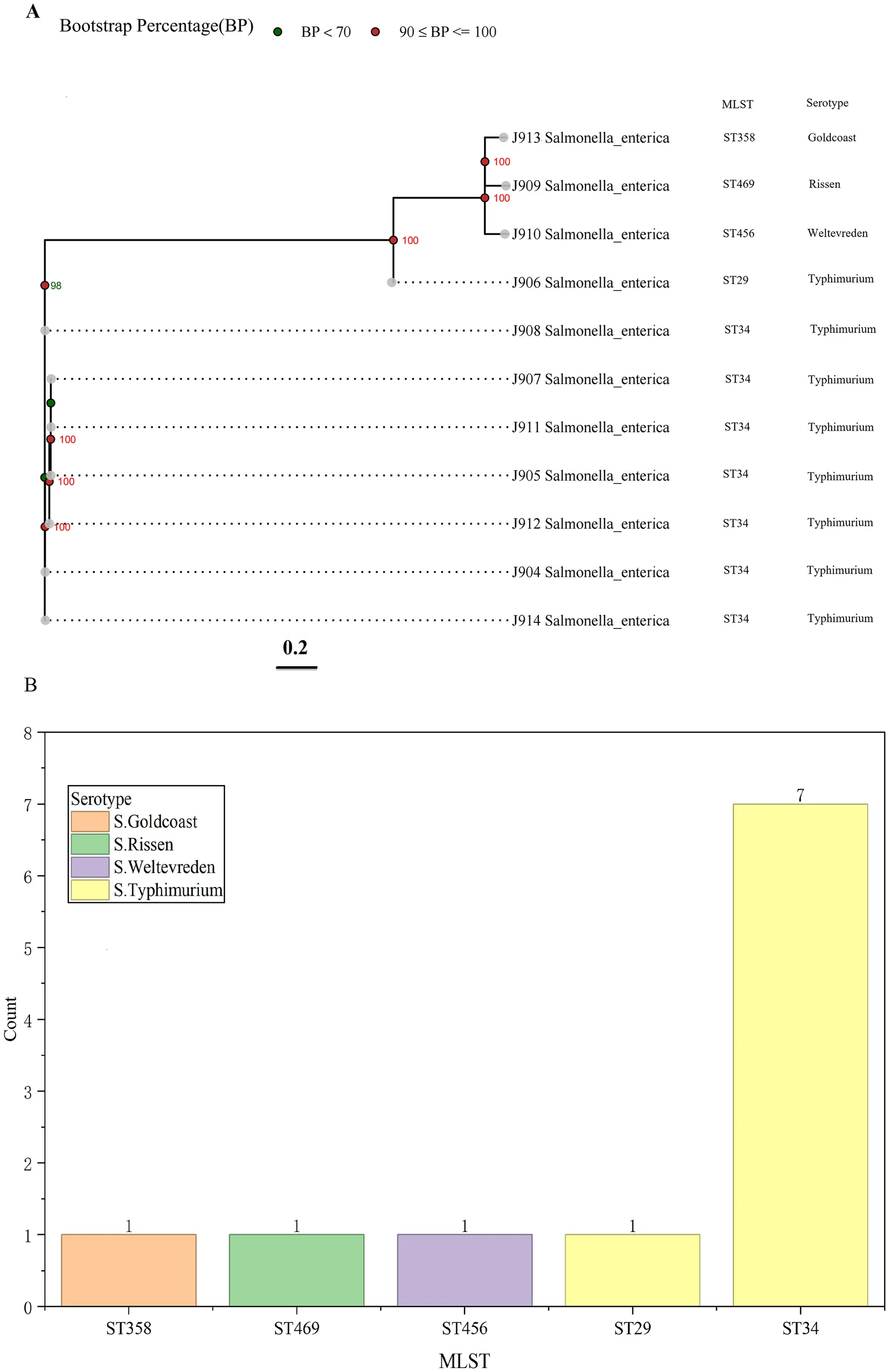
Phylogenetic relationship, MLST and serotype distribution of 11 carbapenem-resistant *Salmonella* isolates. **(A)** Core genome SNP-based maximum-likelihood phylogenetic tree of the 11 sequenced isolates. Node support is indicated by colored circles representing bootstrap percentage **(BP)** values based on 1,000 replicates (green circle: BP < 70; red circle: 90 ≤ BP ≤ 100). The scale bar at the bottom indicates 0.2 nucleotide substitutions per site. Salmonella enterica serotypes and MLST profiles **(STs)** are listed to the right of the tree. **(B)** Distribution and prevalence of STs and their corresponding serotypes among the 11 sequenced isolates, with colors representing different serovars.

The SNP-based maximum-likelihood phylogenetic tree of the core genome illustrated a clear genetic divergence consistent with the MLST profiles and serotyping results (**Figure 6A**). Pairwise SNP analysis revealed that the ST34 isolates exhibited genetic distances ranging from 105 to 702 SNPs, while phylogenetic analysis showed that the isolates clustered into two major lineages (see **Supplementary Figure 2**). One lineage comprised all the *S.* Typhimurium isolates, in which the seven ST34 isolates (J904, J905, J907, J908, J911, J912, and J914) formed a closely related genetic cluster, while the ST29 isolate (J906) branched off adjacent to them. The second major lineage grouped the three non-Typhimurium *Salmonella* isolates (J913, J909, and J910), such that *S.* Goldcoast (ST358) and *S.* Rissen (ST469) share a closer common ancestor (bootstrap value of 100) than *S.* Weltevreden (ST456). Furthermore, the MLST analysis identified five sequence types, with ST34 emerging as the most prevalent in the 11 sequenced isolates (**Figure 6B**). The eight *S.* Typhimurium isolates were distributed into two STs: the majority belonged to ST34 (n=7), while a single isolate was identified as ST29 (n=1). The three non-Typhimurium isolates represented three distinct serotypes and STs: *S.* Goldcoast (ST358, n=1), *S.* Rissen (ST469, n=1), and *S.* Weltevreden (ST456, n=1).

## Discussion

Foodborne salmonellosis represents a critical public health challenge globally. In 2019, foodborne Salmonella was responsible for approximately 1.3 million illnesses and 12,500 hospitalizations, and it remained the leading cause of death in the United States (Scallan Walter et al., 2025). In China, the annual incidence of foodborne salmonellosis was estimated at 245 cases per 100,000 population (Li et al., 2022). In our study, a total of 9,095 fecal specimens were collected, of which 1,746 (19.2%) tested positive for *Salmonella*, highlighting that fecal *Salmonella* carriage remains a significant public health concern in Southwest China.

Our findings reveal a distinct demographic distribution of *Salmonella* infections, characterized by a higher prevalence in males and a sharp concentration in infants and toddlers under 2 years of age. The male-to-female discrepancy observed in this study aligns with historical data from previous studies on enteric pathogens, which frequently report higher rates of *Salmonella* detection among male children (Pokhrel et al., 2023; Yu et al., 2026). One potential hypothesis for the sex-related variation observed in our results is that male toddlers might engage in more active and outdoor-oriented exploratory behaviors, potentially leading to a higher frequency of environmental exposures. However, this behavioral link remains speculative and requires further investigation. Children under two years of age exhibit heightened susceptibility to *Salmonella* infection, owing to their immature immune systems, the introduction of weaning foods that increases exposure to dietary and environmental pathogens, and developmental hand-and-mouth behaviors. Previous studies have similarly demonstrated that the highest incidence of infection occurs in children under two years of age (Yue et al., 2022; Zhou et al., 2024; Yu et al., 2026). Our study underscores the necessity that targeted intervention strategies be implemented for this vulnerable population.

In Southern China, Nanning experiences long, hot, and humid summers, spanning from May to October, with average monthly temperatures frequently exceeding 28 °C and high humidity. The frequency of *Salmonella*, infections in our study is observed throughout the year, but the incidence is particularly high during late spring and summer, with the highest number of cases recorded in May and June. This surge in incidence continues through the summer and early autumn before declining in November. Our findings were concordant with studies conducted in Mali, in Northeastern Poland, in Ghana, and in the Democratic Republic of Congo, where cases occurred throughout the year with increased frequency during peak wet months (Tapia et al., 2015; Tack et al., 2021; Ofori et al., 2023; Fernandez Moreno et al., 2025). We hypothesize that the higher prevalence during late spring and summer could be related to the accelerated proliferation of *Salmonella* in warm environment and a potentially increased risk of exposure via environmental or dietary sources during the humid season. Local CDC departments should place emphasis on maintaining the cold chain for high-risk foods such as meat, poultry, and eggs, and raising public awareness about the hygienic preparation of cold dishes during the humid months.

Serovar analysis indicated that among cases with definitively identified serovars, *S.* Typhimurium predominated, suggesting that this is priority serovars under sentinel surveillance in Guangxi Province. The predominance of this serovar is consistent with previous evidence indicating that *S.* Typhimurium is among the most common serovars responsible for human salmonellosis globally (Xu et al., 2021; Kahsay et al., 2023; Reyes et al., 2025). Nevertheless, the prevalence of *S.* Typhimurium in our study (55.1%) is higher than that reported in other studies conducted in China, such as those by Bacterium-learning Union at 26.6%, Liaoning Province at 28.4%, and Hangzhou at 34.4% (Yue et al., 2020; Wang et al., 2023; Yu et al., 2026). This variation may stem from our specific inclusion criteria, which restricted the study population to children and limited the specimen type solely to fecal samples, unlike other studies with broader patient profiles. Specifically, high pediatric susceptibility is reflected in our age stratification, where *S.* Typhimurium infections remained elevated through the second year of life in both sexes. This vulnerability in toddlers may be due to developmental behaviors. Our data show the dominance of this serovar throughout the year, with a higher prevalence in late spring and summer due to the warm and humid climate.

Over the past decades, antimicrobial resistance in NTS has emerged as a critical global concern, with surveillance data showing overall resistance rates climbing from 20%–30% in the early 1990s to as high as 70% in certain countries by the turn of the century (Su et al., 2004). In the present study, the resistance rate to ampicillin was also found to be high at 70.1%. Additionally, relatively high resistance rates were observed for chloramphenicol (49.1%), sulfamethoxazole (41.0%), minocycline (39.4%), and ceftriaxone (18.8%). Similarly, high resistance rates to ampicillin and sulfamethoxazole have been documented in the United States, Europe, Vietnam, and China (Meakins et al., 2008; Crump et al., 2011; Phu et al., 2022; Li et al., 2024). Despite strict restrictions on the pediatric use of chloramphenicol and minocycline, high resistance rates to these drugs persist. This is likely driven by their historical, intensive application in livestock farming and aquaculture. Supporting this link, prior research has documented elevated resistance to both agents in zoonotic *Salmonella* isolates (Igbinosa, 2015; Fernández Márquez et al., 2017). Third-generation cephalosporins are currently the preferred first-line treatment for pediatric salmonella infections because of the potential adverse effects of fluoroquinolones (Stahlmann and Lode, 1999). This clinical preference likely accounts for the relatively high ceftriaxone resistance rate observed in our study. Notably, *S.* Typhimurium exhibited significantly higher resistance rates than non-Typhimurium serovars across most tested antibiotics, including ampicillin, chloramphenicol, sulfamethoxazole, cefoperazone-sulbactam, minocycline, ceftriaxone, and carbapenems (imipenem/meropenem). Specifically, resistance to ampicillin was alarmingly high at 89.9%, rendering it clinically ineffective. Furthermore, the ceftriaxone resistance rate reached 22.3%, a major public health concern likely driven by the horizontal transfer of ESBL genes (Katsande et al., 2025). This co-resistance to ampicillin and third-generation cephalosporins severely narrows empirical treatment options for pediatric infections, highlighting the urgent need for local antibiotic stewardship. Although carbapenems remained highly active, the detection of resistant *S.* Typhimurium strains (2.6%) requires further monitoring in order to prevent their clonal dissemination in pediatric units.

Our clinical data showed that carbapenem-resistant *Salmonella* infections primarily affected infants and toddlers (aged ≤ 3 years) presenting with gastroenteritis. Although carbapenem resistance poses a severe therapeutic challenge, all 25 patients achieved clinical improvement. This suggests that for localized gastrointestinal infections, clinical outcomes may remain favorable with timely supportive care, regardless of the *in vitro* carbapenem susceptibility status of the isolates. However, the presence of carbapenem-resistant *Salmonella* in pediatric outpatients warns of silent community transmission, necessitating active surveillance to prevent potential invasive outbreaks in young children.

Unlike other Enterobacteriaceae (e.g., *Klebsiella pneumoniae*, *Escherichia coli*, and Enterobacter spp.), carbapenem-resistant *Salmonella* remains rarely isolated from human, animal, or food samples. Nevertheless, five clinically significant carbapenemases—KPC, IMP, NDM, VIM, and OXA-48—have been reported in sporadic *Salmonella* infections globally (Wu et al., 2023). Eight of the 20 carbapenem-resistant *S.* Typhimurium isolates and three of the five carbapenem-resistant non-Typhimurium *Salmonella* isolates were selected for whole-genome sequencing. The results of our study revealed significant abundance and diversity of antimicrobial resistance determinants. Notably, the carbapenemase gene *bla_NDM-5_* was identified in 90.9% (10/11) of the isolates, highlighting its dominant role in conferring carbapenem resistance in this region. This finding is consistent with recent trends that indicate the rapid dissemination of *bla_NDM-5_* among *Salmonella* species (Guo et al., 2026; Qi et al., 2026). Interestingly, we also identified *bla_NDM-13_*​ in a single isolate (J913). As a relatively rare NDM variant previously detected in only three Chinese carbapenem-resistant *Salmonella* isolates, the presence of *bla_NDM-13_*​ suggests ongoing micro-evolution or the introduction of novel resistance alleles within the local bacterial population (Huang et al., 2022; Li et al., 2026; Zhou et al., 2026). Consistent with the antimicrobial susceptibility phenotypes of our carbapenem-resistant *Salmonella* isolates, the co-existence of resistance determinants for phenicols (*floR*), sulfonamides (*sul2*), aminoglycosides (*aac* (Riaz et al., 2025)*-IV*), and tetracyclines (*tet(A)*/*tet(B)*) beyond *β*-lactamase genes further underscores a severe multidrug-resistant profile. Consistent with earlier findings, the co-existence of ESBL genes and multiple other resistance determinants in these carbapenem-resistant *Salmonella* isolates highlights the urgent need for enhanced surveillance and control strategies for antimicrobial resistance (Benamrouche et al., 2025; Dutta et al., 2026; Velmurugan et al., 2026). Therefore, actively monitoring these co-resistance markers is essential, as the presence of non-carbapenem antibiotics can exert selective pressure that favors the maintenance of carbapenem-resistant plasmids.

Plasmid replicon typing further elucidated the potential dissemination pathways of these resistance genes. In this study, a specific cluster of seven isolates was found to carry both IncHI2 and IncHI2A replicons, with ST34 *S.* Typhimurium being the predominant sequence type. This finding is consistent with previous reports indicating that the majority of ST34 *S.* Typhimurium strains harbor IncHI2 plasmids sharing similar backbone sequences (Deng et al., 2024). These observations point to either the clonal expansion of a single resistant lineage or the horizontal transfer of a shared IncHI2-type plasmid. However, the coexistence of *bla_NDM-5_*, IncHI2, and IncHI2A in a single *Salmonella* genomic background is exceedingly rare, consistent with the few studies that have reported *bla_NDM--_*positive IncHI2 *Salmonella* strains (Li et al., 2025; Yang et al., 2026). Given that IncHI2 plasmids possess cross-species transmission capabilities—spanning *Enterobacter* spp. (*E. hormaechei* and *E. cloacae*), *Salmonella* spp., *Citrobacter freundii*, and *Leclercia adecarboxylata*—Our findings highlight that these mobile elements may serve as key vehicles driving MDR phenotypes in Salmonella (Liu et al., 2026). A prior study also demonstrating that the IncHI2 plasmid has superseded the IncX3 plasmid as the primary vector mediating *bla_NDM-5_* transmission on certain farms (He et al., 2023). Notably, genomic analysis revealed that while strain J913 (*bla_NDM-13_*) carried its resistance gene on a fully conjugative IncI1-I(Alpha) plasmid contig possessing all key transfer components (oriT, relaxase, T4CP, and T4SS), the *bla_NDM-5_* genes in the remaining ten isolates resided on contigs devoid of detectable plasmid replicons or conjugative machinery. These findings indicate that the *bla_NDM-5_* genes in these isolates are either chromosomally integrated or situated on non-mobilizable elements, or remain unresolved owing to the constraints of short-read genome assembly. Consequently, further investigations, such as long-read sequencing and conjugation assays, are warranted to elucidate the precise transmission routes and mobilization mechanisms of these *bla_NDM-5_* genes.

*Salmonella* colonizes the intestinal epithelium, subsequently invading the host cells to release enterotoxins that ultimately trigger clinical pathogenesis (Dos Santos et al., 2019). Numerous critical virulence factors of *Salmonella* are harbored within *Salmonella* pathogenicity islands (SPIs) (Adesiji et al., 2025). Consistent with this pathogenic framework, our genomic analysis revealed a highly conserved virulence profile among the eleven carbapenem-resistant Salmonella strains. Specifically, 83 core virulence genes—predominantly involved in adhesion, invasion, and secretion systems—were present in 100% of the isolates, with this high degree of conservation ensuring the functional stability of their virulence. Notably, key loci within SPI-1 and SPI-2 encode Type III secretion systems 1 and 2 (T3SS-1 and T3SS-2), respectively (Jiang et al., 2021). SPI-1 is intrinsically linked to every stage of intestinal epithelial cell invasion, intracellular replication, and colonization in human cells (Saleh et al., 2023). Conversely, SPI-2 is associated with oral infection, the promotion of intestinal inflammation, and systemic dissemination from the gut, thereby facilitating the successful colonization of *Salmonella* within the host (Pospíšilová et al., 2025). The *mgtCB* operon plays a vital role in bacterial growth and replication; specifically, the *mgtC* and *mgtB* genes encode a Mg²^+^ transporter that strictly regulates intracellular magnesium homeostasis (Alix and Blanc-Potard, 2008). Importantly, mig-14 may contribute to polymyxin B resistance in *Salmonella* by reducing outer membrane permeability and promoting biofilm formation (Sheng et al., 2019). This finding implies that the acquisition of polymyxin B resistance in carbapenem-resistant *Salmonella* strains would pose a formidable challenge to clinical treatment strategies. This near-universal conservation of determinants essential for host entry and intracellular survival indicates that despite genetic variations, these isolates maintain an uncompromised baseline potential to colonize and invade hosts.

Globally, *S.* Typhimurium ST34 has emerged as a predominant epidemic clone driving the dissemination of multidrug resistance (Luo et al., 2023; Supa-Amornkul et al., 2023). Although the *bla_NDM-5_*_-_bearing *S.* Typhimurium ST34 lineage has been documented in China, this lineage constituted the predominant genotype in our cohort, with the seven isolates forming a closely related genetic cluster (Deng et al., 2024; Yang et al., 2026). Interestingly, our finding contrasts with a previous study in South China (covering Guangdong, Guangxi, and Hong Kong), which identified the clonal dissemination of *bla_NDM-1_*-positive ST34 *S.* Typhimurium (Deng et al., 2024). While this discrepancy suggests a possible evolutionary shift in carbapenemase variants within the regional ST34 clone, the tight genomic clustering of these isolates indicates a recent clonal expansion from a common origin, which underscores its potential for rapid localized transmission. These observations also indirectly imply that this resistant clone or plasmid may already be present in pediatric populations across multiple regions in China; however, the precise transmission pathways remain to be validated by future multicenter genomic surveillance. Considering the established role of *S*. Typhimurium ST34 as a major foodborne pathogen, the integration of *bla_NDM-5_* into this successful genetic background may facilitate its dissemination to humans, potentially through foodborne transmission pathways. This highlightsthe critical need for implementing systematic genomic surveillance across the human-animal-environment interface to intercept the spread of this high-risk clone.

The current study had some limitations. First, this was a single-center study conducted over a one-year period, which may limit the generalizability of our findings. Future multi-center studies covering a broader temporal span are needed to validate our results. Secondly, we did not follow up with the patients, which restricted our ability to evaluate the clinical outcomes and long-term prognosis of these carbapenem-resistant *Salmonella* infections. Thirdly, WGS was performed on only a subset (11/25) of the carbapenem-resistant isolates. While efforts were made to select these 11 isolates based on their temporal distribution, patient age diversity, and clinical settings, excluding the other 14 isolates remains a limitation that may restrict a comprehensive depiction of the genomic diversity of carbapenem-resistant *Salmonella* in our institution. Given the limitations of Illumina short-read sequencing in resolving plasmid structures, the observed dominance of this *bla_NDM-5_*-carrying ST34 lineage should be interpreted with caution, necessitating future long-read sequencing, conjugation assays, or broader genomic surveillance to definitively confirm the physical localization of *bla_NDM-5_* on IncHI2 plasmids. Lastly, a limitation of this study is the lack of direct comparative genomic analysis with publicly available national or international *bla_NDM-5_*-positive *S.* Typhimurium ST34 genomes. While our results demonstrate a high prevalence of this lineage within our healthcare facility, further studies incorporating broader domestic and global reference genomes are warranted to delineate the evolutionary trajectory and reconstruct the precise transmission dynamics of these isolates.

## Conclusion

In conclusion, this study characterizes the epidemiology, resistance profiles, and genomic landscapes of pediatric Salmonella isolates, identifying *S*. Typhimurium as the primary causative agent. The strain isolation rate showed a male predominance, a sharp concentration in infants and toddlers under 2 years of age, and a distinct seasonal peak during late spring and summer. High phenotypic resistance rates were observed to ampicillin, chloramphenicol, and sulfamethoxazole, underpinned by a high diversity of antimicrobial resistance determinants. Crucially, genomic analysis revealed a highly conserved virulence profile among the sequenced carbapenem-resistant isolates. Furthermore, the *bla_NDM-5_* carbapenemase gene was the predominant resistance determinant, predicted to be mainly associated with IncHI2 and IncHI2A plasmid replicons within the ST34 *S.* Typhimurium lineage. Although the *bla_NDM-5_*-bearing *S.* Typhimurium ST34 lineage has been documented in China, its identification as a closely related genetic group among the sequenced isolates in this pediatric cohort suggests a potential for clonal dissemination, highlighting the need for strengthened genomic surveillance in Nanning, China.

## Data Availability

The datasets presented in this study can be found in online repositories. The names of the repository/repositories and accession number(s) can be found below: https://www.ncbi.nlm.nih.gov/, PRJNA1490610.
